# Correction to “Chemical and Structural Trends on Stability and Electronic Properties of CsMX_3_ (M = Ge, Sn, or Pb; X = F, Cl, Br, or I) Inorganic Perovskite Polymorphs Including Relativistic and Quasiparticle Effects”

**DOI:** 10.1021/acs.jpcc.6c05760

**Published:** 2026-10-08

**Authors:** Débora Nazaré de Freitas, Claudio R. da Silva, Jeanlex S. de Sousa, Lara K. Teles, Marcelo Marques

We have identified a technical error in our original article that led to an incorrect interpretation of the stability of the δ-CsSnI_3_ perovskite phase. The error originated during the construction of the atomic position file (POSCAR). Although the incorrect structures converged within the adopted convergence criteria, a posteriori analysis confirmed that they exhibited the orthorhombic symmetry (space group *Pnma*) characteristic of the δ (yellow) phase. As a result, the δ phase was erroneously identified as the least stable tin-iodide compound, contradicting results previously reported in the literature. At the time, we were unable to explain why this phase did not exhibit near-degeneracy with respect to the γ phase, as expected. However, with the corrected implementation, both phases are now found to be nearly degenerate and competitive in stability.

Specifically, our revised calculations show that the δ-*Pnma* phase is now the most stable, closely competing in stability with the γ-*Pnma* phase. The cohesive energy difference per formula unit, previously reported as 0.34 eV, has been recalculated to only 0.01 eV. Consequently, Tables 1 (structural parameters) and 2 (cohesive energies) have been updated, as presented in this erratum. Similarly, Figure 3 of the original paper should be replaced by Figure 3 below. In addition, the fundamental band gap has slightly increased from 2.71 to 2.97 eV, and the results reported for δ-CsSnI_3_ in Table 3 of the original paper must be updated according to the values presented in Table 3 here. For simplicity, only the updated values for the δ phase are included, while all other values from the original table remain unchanged. It is important to emphasize that these corrections do not affect the overall conclusions of the original article.

**Table 1:** Corrected Lattice Parameters *a*, *b*, and *c* (in Å), Angles *α*, *β*, and *γ* (in deg), and Volume (in Å^3^/f.u.) for *δ*-CsSnI_3_ ^a^

CsSnI_3_	*a*	*b*	*c*	α	β	γ	Vol.f.u.
δ-*Pnma*	4.83 (4.66)	10.78 (10.10)	18.13 (17.18)	90.00	90.00	90.00	235.84 (242.97)

a For clarity, previously reported results obtained using the incorrect structure are given in parentheses.

**Table 2:** Corrected Cohesive Energy (in eV per Formula Unit) for the *δ*-CsSnI_3_ Compound Alongside Other Phases Reported in the Original Paper^a^

	*Pm*3̅*m*	*P*4/*mbm*	*I*4/*mcm*	γ-*Pnma*	*R*3̅*m*	*δ*-*Pnma*
CsSnI_3_	–14.760	–14.779	–14.779	–14.765	–14.488	–14.807 (−14.456)

a The value previously obtained using the incorrect structure is shown in parentheses for clarity.

**Figure 3:**
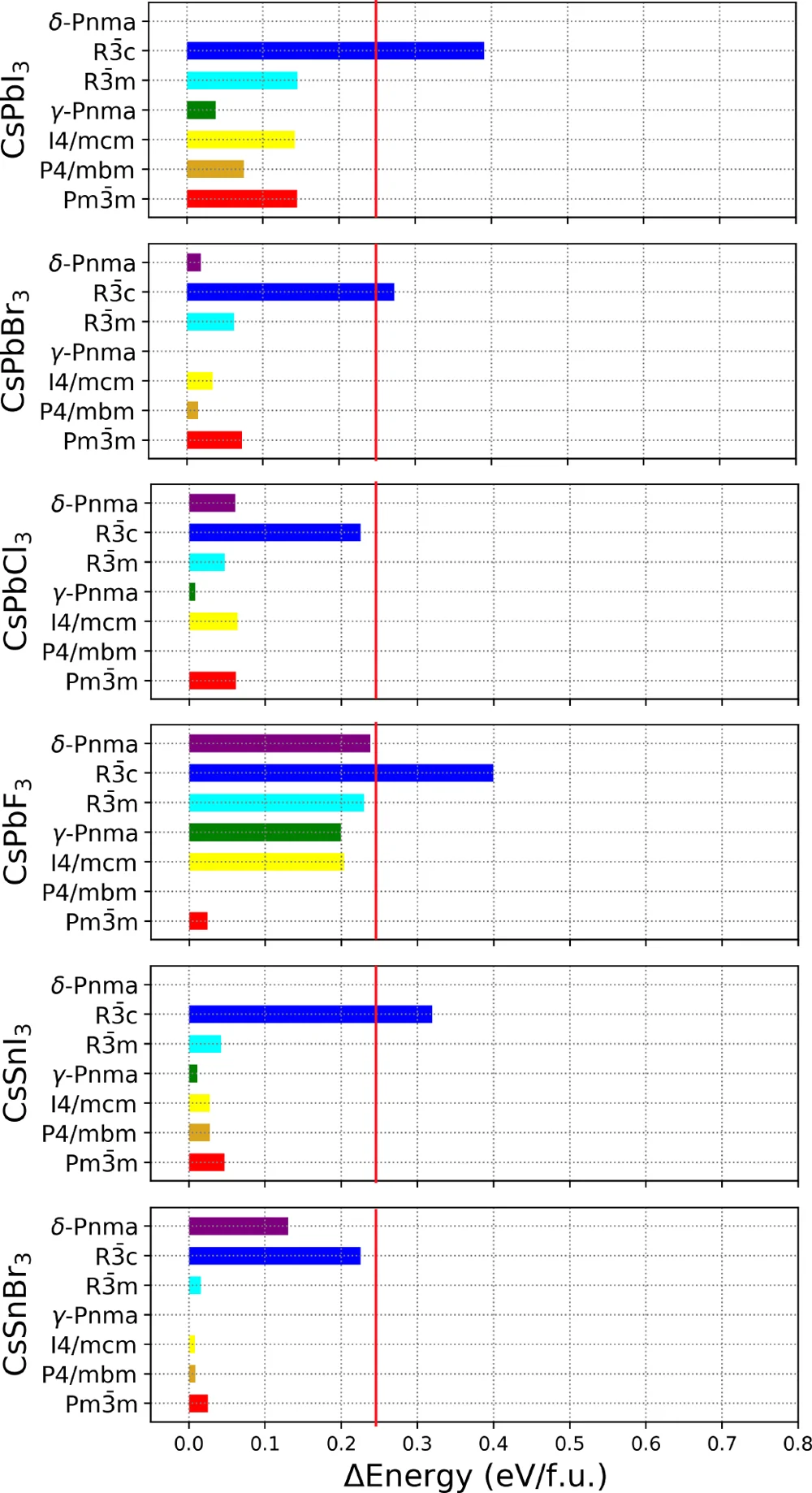
Difference in cohesive energy (in eV/f.u.) between each structure and the ground-state structure of the corresponding compound. The absence of a bar indicates the most stable structure for each compound. The red vertical line marks the approximate energetic stability threshold.

**Table 3:** Fundamental Band Gap Values for the Corrected *δ*-CsSnI_3_ Phase Calculated in This Work^a^

CsSnI_3_	*Pm*3̅*m*	*P*4/*mbm*	*I*4/*mcm*	γ-*Pnma*	*R*3̅*m*	*R*3̅*c*	δ-*Pnma*
DFT-1/2+SOC*	0.96	1.17	1.16	1.35	2.43 (2.42)	1.82	3.03 (2.97)
						(1.81)	[2.82 (2.71)]
Experimental	1.23			1.21–1.31, 1.30			
				1.32, 1.31			
DFT-1/2+SOC	0.92, 0.96			1.36, 1.34			
	1.02, 0.82						
HSE	0.59, 0.87						
HSE+SOC	0.49			1.1, 0.94			
HSE06	0.74, 1.15	0.91, 0.60		1.24, 1.49, 1.13			2.69
	0.69	0.92					
HSE06+SOC	0.30, 0.82	1.03, 0.71		1.12, 0.50			2.76
	0.34	0.45		1.13, 0.80			
GW	1.37						
G_0_W_0_	0.82						
QSGW	1.21, 1.35	1.49, 1.494		1.5			
	1.354						
QSGW+SOC	1.01, 1.008	1.29, 1.288		1.30			2.70

a Reference values and their corresponding literature citations remain unchanged from the original article. The asterisk (*) denotes the model developed in this work. Indirect gaps are indicated in parentheses, while previous values obtained using the incorrect structure are shown in brackets for comparison.

